# Spotlight on drug-resistant *Shigella*: raising awareness within general practice

**DOI:** 10.3399/bjgp23X732537

**Published:** 2023-03-31

**Authors:** Hannah Charles, Hannah McCall, Alison Mason, Kirsty Foster, Richard Ma, Claire Jenkins, Katy Sinka, Gauri Godbole

**Affiliations:** UK Health Security Agency, London.; UK Health Security Agency, London.; Brunswick Park Medical Practice, London.; UK Health Security Agency, London.; Imperial College London, London.; UK Health Security Agency, London.; UK Health Security Agency, London.; UK Health Security Agency, London.

## WHAT IS SHIGELLOSIS?

Shigellosis is a bacillary dysentery caused by *Shigella*, resulting in mild to severe diarrhoea (sometimes containing blood), stomach cramps, anorectal pain, nausea, vomiting, and fever. Symptoms are typically seen 1 to 4 days after exposure and are commonly mistaken for food poisoning.^1^

*Shigella* is highly infectious. Transmission occurs via the faecal–oral route through direct contact with an infected person, or exposure to contaminated surfaces, objects, food, or water.^1^

Historically, most cases of shigellosis in the UK were associated with travel to endemic countries. Over the past 10 years, non-travel-associated cases have risen to account for a large proportion of all cases reported among adults.^2^ Cases in males are now increasingly associated with sexual transmission via direct oral‒anal contact, or oral sex after anal sex or play, including fingering or use of sex toys. In 2019, 52% of adult cases were thought to be sexually transmitted among gay, bisexual, and other men who have sex with men (GBMSM).^2^ Following a decline in reported diagnoses in 2020, likely due to impacts on healthcare access and behaviour change, diagnoses among GBMSM have increased in 2021 to pre-COVID-19 pandemic levels.^2^

## WHY IS SHIGELLOSIS A PROBLEM NOW?

The number of cases of sexually transmitted *Shigella* spp. among GBMSM has nearly doubled in the last decade,^2^ and there are multiple strains with a variety of multidrug-resistant profiles circulating in these sexual networks.^3^

The UK Health Security Agency is responding to an outbreak of extensively drug resistant (XDR) *S. sonnei* mainly affecting GBMSM,^4^ where cases began to increase rapidly from September 2021. Characteristics of outbreak cases are summarised in Box 1. As of 3 October 2022, there have been 150 confirmed cases within this outbreak in England.^5^

**Box 1.** Characteristics of cases within ongoing *S. sonnei* outbreak^6^

Median age of 34 years. Living in London (65%) — yet cases have been identified from all regions in England. HIV negative and taking HIV pre-exposure prophylaxis (74%). Linked to high-risk sexual networks, including attendance at chemsex parties and sex-on-premises venues. Part of a single cluster, which is extensively drug resistant, including to ceftriaxone.

## HOW IS THE MANAGEMENT DIFFERENT?

While antibiotic treatment is not required for all cases of shigellosis, cases with prolonged symptoms or complications may require antibiotics. Among GBMSM, the proportion of all *Shigella* spp. isolates that are multi-drug resistant, or XDR is very high, often exceeding 90%.^2^ The current XDR strain of *S. sonnei* is of particular concern as it displays resistance markers against many commonly used antibiotics (macrolides, fluoroquinolones, aminoglycosides, sulphonamides, trimethoprim, tetracycline, and third-generation cephalosporins). This means that first-line agents will *not* be effective for treatment for this XDR strain and there is the potential for treatment failures.^6^ There are concerns of secondary transmission to the wider non-GBMSM population, and transfer of XDR genes to other *Shigella* species, or to other gastrointestinal bacteria.

### What should be done?

Clinicians managing cases of community-acquired diarrhoea should be aware that shigellosis should be considered in adult males where sexual transmission is a possible route of acquisition. GBMSM may not be aware of the risk of sexual transmission and may not volunteer their recent sexual history unless specifically asked.

Clinicians should specifically ask for travel and sexual history for those presenting with acute diarrhoeal illness, and *request appropriate diagnostic tests*, that is, faecal bacterial culture and polymerase chain reaction test (if available), and antibiotic susceptibility testing for *Shigella* spp., from adult males presenting with acute diarrhoea who have not travelled and identify as GBMSM. It is also recommended that stool samples be taken from those with prolonged diarrhoea (>7 days), blood in stools, and those with severe illness requiring hospitalisation (Box 2).^7^^,^^8^

**Box 2.** Clinical risk assessment

**Key questions for all suspected cases**	**Risk groups for transmission of gastrointestinal pathogens^7^**
• *Timeline*: When did symptoms start and when did they resolve?	A Any person unable to perform adequate personal hygiene because of capacity or lack of access to hygiene facilities, for example, disabled or homeless patients
• *Travel history*: Did the case travel abroad or within the UK, during the incubation period?	B All children aged 5 years old or under (up to 6th birthday)
• *Contacts at risk*: Consider all household contacts,^a^ *and* sexual contacts of GBMSM	C Food-handlers, including bar workers and waiters
• *Onward transmission*: Is the case in a risk group for transmitting gastrointestinal infections?	D Healthcare workers, including clinical, social care, or nursery staff

a *Household contact = someone who lives, or has stayed overnight, in the same household as the case and/or has shared a bathroom and/or eaten food prepared by the case while the case was symptomatic and up to 48 hours after symptoms ceased. GBMSM = gay, bisexual, and other men who have sex with men.*

### What is the treatment?

The key message is that effective antibiotic treatments are limited for this extremely resistant strain.

The GP should discuss suspected or confirmed cases with their local microbiologist and always send a stool sample before commencing treatment.^7^^,^^8^

### What should GPs advise patients?

Hygiene measures remain the mainstay for the prevention of onward transmission. Suspected and confirmed cases of shigellosis should wash their hands regularly, before preparing or eating food, and after using the toilet. Practising good sexual hygiene is also important to reduce transmission, and suspected or confirmed cases should additionally be encouraged to avoid oral–anal sexual contact until 1 week after diarrhoea has resolved.^7^ GBMSM with shigellosis may be at risk of other sexually transmitted infections, including HIV, and should be referred to a sexual health clinic for full sexually transmitted infection and HIV testing and management, to ensure that all sexual health needs are met.

### Further actions: notification

Cases of suspected or confirmed acute shigellosis should be notified promptly to the local Health Protection Team as usual (https://www.gov.uk/health-protection-team).
